# Butyrate Glycerides Protect against Intestinal Inflammation and Barrier Dysfunction in Mice

**DOI:** 10.3390/nu14193991

**Published:** 2022-09-26

**Authors:** Haidong Wang, Haohan Chen, Yueying Lin, Geng Wang, Yanqiu Luo, Xinyu Li, Minqi Wang, Mingyan Huai, Lily Li, Adriana Barri

**Affiliations:** 1 The Key Laboratory of Molecular Animal Nutrition, Ministry of Education, College of Animal Sciences, Zhejiang University, Hangzhou 310058, China; 2 BASF (China) Co., Ltd., Shanghai 200137, China; 3 BASF SEA Pte Ltd., Singapore 038987, Singapore; 4 BASF SE, 68623 Ludwigshafen, Germany

**Keywords:** butyrate glycerides, intestinal barrier, intestinal inflammatory, brush border, MAPKs

## Abstract

This study investigates the attenuating effects of butyrate glycerides (BG) on intestinal inflammatory responses and barrier dysfunction induced by LPS stimulation. An initial dose-response test was carried out to identify the optimal dose of BG for further testing. The mice were given intragastric administration of BG at different doses followed by lipopolysaccharide (LPS) intraperitoneal injection. The small intestinal morphology and cytokine mRNA expression were measured. With 1.5 g/kg BW BG administration, it was possible to alleviate the injury of duodenal morphology, attenuate ileum villus height reduction and promote IL-10 mRNA expression. Therefore, the optimal dosage of 1.5 g/kg BW BG was selected for the main experiment. The ultrastructure image of jejunum and ileum epithelial cells, mRNA expression, the level of cytokine and immunofluorescence in the ileum were analyzed. The results showed that BG maintain the ileac brush border, tight junction structures and protein expression. BG attenuated the increased inflammatory cytokines, TLR4 and JNK mRNA expression. Taken together, 1.5 g/kg BW BG administration maintained intestinal barrier function and reduced intestinal and body inflammation responses induced by LPS in mice. The mechanism by which BG alleviated intestinal inflammatory response and maintained intestinal barrier function may be related to the JNK signaling pathway.

## 1. Introduction

The intestinal tract is the largest immune organ, and the small intestine is the main part where the digestion and utilization of nutrients take place [1]. Intestinal epithelial cells form physical and biochemical barriers in the intestinal tract, and play a crucial role in protecting against toxic substances and pathogenic microorganisms [2]. The brush border consists of neatly arranged microvilli of intestinal epithelial cells, which work as an intestinal physical electrostatic barrier against pathogenic microorganisms [3,4]. For a complete intestinal epithelial cell layer, the paracellular pathway of epithelial cells remains impermeable, and molecular structures such as tight junction (TJ) structures between cells determine the permeability of the paracellular pathway [5]. Pathogen infections cause diarrhea, leading to morbidity and mortality in children and infants, and symptomatic episodes increase the risk of chronic diseases [6,7]. Moreover, intestinal barrier dysfunction leads to increased intestinal permeability and imbalance of intestinal homeostasis in animals. These symptoms are prevalent in patients with inflammatory bowel disease (IBD) [8,9].

Short-chain fatty acids (SCFAs) including acetic acid, propionic acid, and butyric acid, are mainly produced by *lactobacillus*, *bifidobacteria* and other beneficial bacteria in the large intestine and they ferment indigestible carbohydrates such as dietary fiber, resistant starch and oligosaccharides [10]. Amongst the SCFAs, butyric acid has attracted wide attention because of its biological function in providing energy for intestinal epithelial cells in the colon, regulating intestinal gene expression and suppressing intestinal inflammation [11,12,13].

The use of free butyric acid is limited due to its unpleasant smell, short half-life and other obstacles. Butyrate glycerides (BG) are formed by the esterification of butyric acid to glycerol. Butyric acid glycerides have no irritating odor. Monobutyrin can be utilized directly and is of benefit to intestinal epithelial cells [14]. Moreoever, BG gradually releases butyric acid and is digested to monobutyrin in the intestine by intestinal lipase hydrolysis [15,16]. Butyric acid glycerides are known to improve intestinal barrier function in rats [17,18,19]. In addition, monobutyrin is known to inhibit intestinal pathogens and promote the growth of beneficial bacteria in the intestine [20,21].

As far as we know, there are few studies on BG in protecting intestinal barrier function under the conditions of intestinal inflammation and barrier dysfunction. Therefore, this study was carried out by constructing these pathological intestinal conditions using an lipopolysaccharide (LPS) challenging to investigate the effects of BG for the protection of intestinal inflammation and barrier injury. The obtained results provide a valuable reference for BG application in maintaining intestinal health for animals. 

## 2. Materials and Methods

### 2.1. Animals and Experimental Treatment

BG was kindly provided by BASF (Shanghai, China), as the product Silo Health 104 liquid. The approximate composition of BG is 55–62% monoglycerides and diglycerides of butyric and 38–48% free glycerol. All animal experiments were approved by the Institutional Animal Care and Use Committee of Zhejiang University (Approval number: ZJU20210237).

#### 2.1.1. Animal Experiment 1

**Figure 1:**
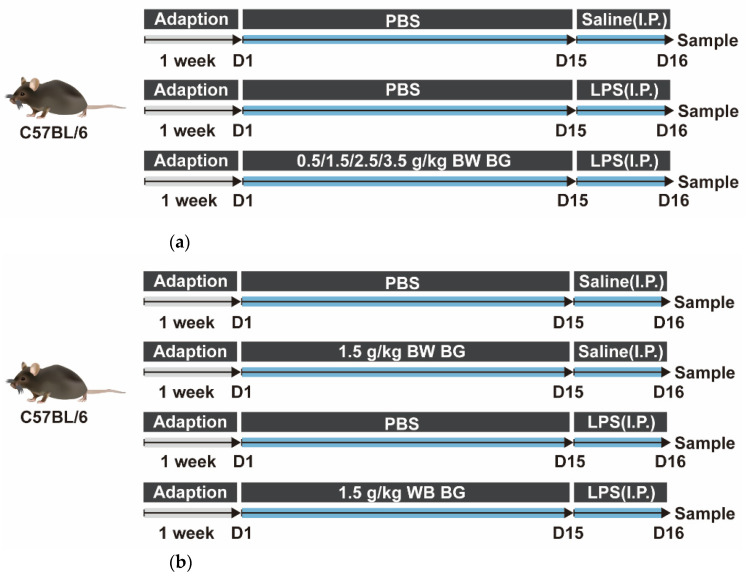
Schematic for the experimental design. (**a**) Dosage screening trial of BG for alleviating intestinal inflammation and barrier dysfunction in LPS-stimulated mice; (**b**) Effects of 1.5 g/kg BW BG on intestinal inflammation and barrier dysfunction in mice challenged by LPS.

A total of 60 healthy male C57BL/6 mice four weeks old were provided by Zhejiang Academy of Medical Sciences (Hangzhou, China), and standard rations were supplied by the Experimental Animals Center of Zhejiang University. After one week of adaption, the mice were intragastrically administered 0.4 mL phosphate buffer saline (PBS) or 0.5, 1.5, 2.5 or 3.5 g/kg BW BG once a day for 14 consecutive days. On the 15th day, the mice administrated with BG were intraperitoneally injected (I.P.) with 10 mg/kg BW LPS, while the mice with PBS intragastric administration were intraperitoneally injected with saline or 10 mg/kg BW LPS. All mice were allowed free access to food and water and housed in plastic cages located in an air-conditioned room with the temperature set at 22 ± 3 °C, relative humidity: 50 ± 10% and light/dark cycle: 12 h/12 h. The body weights of every mouse was collected weekly. After 24 h, blood samples were collected from the retro-orbital plexuses, then centrifuged at 3000× *g* for 10 min to isolate serum and stored at −80 °C. The mice were euthanized with pentobarbital sodium. The 2–3 cm length intestinal segments near the pylorus of the stomach, in the middle of the small intestine, and near the caecum were identified to be duodenum, jejunum and ileum accordingly. Sections of middle duodenum, middle jejunum and distal ileum were isolated and stored at −80 °C and further analyzed with enzyme-linked immunosorbent assays (ELISA) and quantitative polymerase chain reaction (q-PCR). Part of the intestinal segments (3–5 mm length) were flushed gently with cold PBS twice for histopathological analyses. The classification and experimental design of the treatments are shown in Figure 1a.

#### 2.1.2. Animal Experiment 2

A total of 24 4-week-old healthy male C57BL/6 mice were provided by Zhejiang Academy of Medical Sciences (Hangzhou, China). After one week of adaption, the mice were randomly divided into four groups and treated as follows: PBS+Saline group (intragastric administration with PBS, saline intraperitoneal injection on the 15th day), BG+Saline group (intragastric administration with 1.5 g/kg BW BG, saline intraperitoneal injection on the 15th day), PBS+LPS group (intragastric administration with PBS, LPS intraperitoneal injection on the 15th day) and BG+LPS group (intragastric administration with 1.5 g/kg BW BG, LPS intraperitoneal injection on the 15th day). The housing conditions and sampling methods of the mice were the same as described previously in experiment 1. The classification and experimental design of the treatments are shown in Figure 1b. Twenty-four hours after LPS or saline injection, the mice were anesthetized to death. Then the serum samples, the middle duodenum, proximal jejunum and terminal ileum were collected as described in experiment 1.

### 2.2. Enzyme-Linked Immunosorbent (ELISA) Assay

The content of cytokines such as IL-1β TNF-α, IL-6, and IL-10 in serum and the ileum homogenates were determined by ELISA kit (MLBIO Biotechnology, Shanghai, China) according to the manufacturer’s instructions. The protein concentration was detected by the bicinchoninic acid assay kit (BCA, KeyGen Biotech, Nanjing, China).

### 2.3. Intestinal Morphology Analysis

The duodenum, jejunum and ileum sections were fixed in 4% paraformaldehyde (PFA) for 24 h. After fixing, the tissues were dehydrated with an ethanol gradient and embedded in paraffin. Then the tissue specimens were cut into slices of 6 μm thickness and stained with hematoxylin and eosin (H&E). A microscope (Olympus CX21, Tokyo, Japan) was used to obtain the intestinal morphology. Then, the villus height and crypt depth were measured by Image-Pro Plus software (IPP, Media Cybernetics, Inc., Rockville, MD, USA) and the villus height/crypt depth was calculated.

### 2.4. Microvilli and Tight Junction Structure Observation by SEM and TEM

The intestinal tissue samples of jejunum and ileum for transmission electron microscope (TEM) analysis were fixed in 2.5% glutaraldehyde solution for 24 h. Then the samples were treated with 1% Osmic acid solution for 1 h. The intestinal samples were dehydrated with gradient concentration ethanol for 15 min each (30%, 50%, 70%, 80%, 90% and 95%), and 100% ethanol for 20 min. Then the intestinal samples were treated with 100% acetone and spurr resin mixture (1:1 for 1 h and 1:3 for 3 h). Finally, the intestinal samples were treated with a pure spurr resin mixture for 12 h. The specimen was heated at 70 °C for 12 h. The embedded samples were sliced (70–90 nm) using an ultramicrotome (Leica Em Uc7, Leica, Wetzlar, Germany) and stained with uranyl acetate and alkaline lead citrate. Lastly, the intestinal tissue samples were evaluated with the Hitachi Model H-7650 electron microscope (Hitachi, Tokyo, Japan).

The intestinal tissue samples of jejunum and ileum for scanning electron microscope (SEM) analyses were fixed in 2.5% glutaraldehyde solution for 24 h and fixed with 1% Osmic acid solution for 1 h. The intestinal samples were dehydrated with gradient concentration ethanol solution (30%, 50%, 70%, 80%, 90%, 95%) for 15 min each procedure. Lastly, the samples were treated with 100% ethanol twice for 20 min each time. The samples were dehydrated in a critical point dryer and coated with gold–palladium. Finally, the specimen was observed and photographed with Hitachi Model SU-8010 SEM (Hitachi, Tokyo, Japan).

Two to four measurements of five cells per sample were used to determine the microvilli length and rootlet density. Microvilli length and rootlet density were measured by Image-Pro Plus software (IPP, Media Cybernetics, Inc., Rockville, MD, USA).

### 2.5. Quantitative Real-Time PCR

The total RNA of the ileum samples was extracted using the TRIzol Reagent (Invitrogen, Carlsbad, CA, USA). RNA purity and concentration were determined by a NanoDrop 2000 spectrophotometer (Thermo Fisher Scientific, Waltham, MA, USA). Complementary DNA (cDNA) was synthesized from 1 μg of total RNA using a MonScript^TM^ RTIII kit (Monad, Wuhan, China) following the manufacturer’s protocol. qPCR analyses were performed with MonAmp^TM^ SYBR^®^ Green qPCR Mix (Monad, Wuhan, China). The RT-qPCR application was performed in triplicate using a CFX96™ qPCR system (Bio-Rad, Hercules, CA, USA). The following amplification protocol was used: 95 °C for 30 s, followed by 40 cycles (95 °C for 10 s, 60 °C for 30 s and 72 °C for 20 s). The mRNA expression of target genes was analyzed with the 2^−ΔΔCt^ method, and β-actin was used as a housekeeping gene to normalize the mRNA expression of the target gene. Primer sequences were synthesized in Tsingke (Beijing, China) and are listed in Table 1.

### 2.6. Immunofluorescence Analysis

The detection and localization of ZO-1, claudin-1 and occludin in the mice ileum tissue was analyzed by immunofluorescence. The mice ileum tissue was fixed with 4% PFA, embedded in paraffin and then sliced. The section of ileum tissue was deparaffinized and rehydrated by treating with xylene and gradient ethanol. The tissue slides were blocked with 3% BSA at room temperature for 30 min, incubated with ZO-1, claudin-1 and occludin (Servicebio, Wuhan, China, dilution 1:500) primary rabbit antibodies overnight at 4 °C. After incubating with secondary antibody and counterstaining with 4, 6-diamidino-2-phenylindole (DAPI, Servicebio, G1012), the digital images of sample slides were obtained by a fluorescence microscope (Nikon Eclipse C1, Nikon, Tokyo, Japan). The fluorescence intensity was measured by ImageJ software (National Institutes of Health, Bethesda, MA, USA).

### 2.7. Statistical Analysis

Statistics were analyzed by SPSS 20.0 statistical software (SPSS Inc., Chicago, IL, USA). All data were presented as mean ± standard error of the mean (SEM). A one-way ANOVA with Tukey’s multiple comparisons tests among groups was performed. A two-tail unpaired *t*-test was conducted to determine the differences between the two groups. *p* values < 0.05 were considered statistically significant.

## 3. Results

### 3.1. Growth Performance

**Figure 2:**
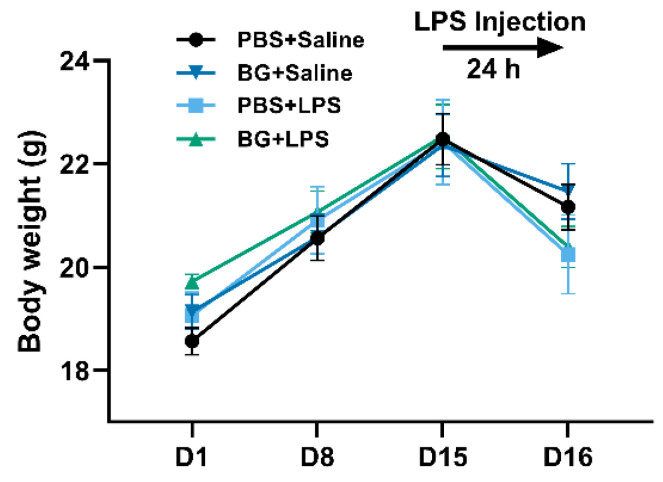
Changes of body weight during the experiment. PBS+Saline, mice administered PBS and injected with saline; BG+Saline mice administered 1.5 g/kg BW BG and injected with saline; PBS+LPS, mice administered PBS and injected with LPS; BG+LPS, mice administered 1.5 g/kg BW BG and injection with LPS. Data are presented as mean ± SEM, *n* = 6. An unpaired *t*-test was used for statistical analysis between two groups.

An amount of 1.5 g/kg BW BG administered had no significant effects on the growth performance of mice (*p* > 0.05, Figure 2). Moreover, no significant differences in body weight were observed between the control and the other three BG dosage treatments during the experimental period, which suggests that BG administration varying from 0.5 to 3.5 g/kg BW has no negative effects on mice growth (shown in Figure S1). A previous study reported that monobutyrin at 0.25% (*w/v*) added to the drinking water of mice for 6 weeks did not affect body weight [19]. Nguyen et al. indicated that monobutyrin supplementation (0.25–1.5 g/100 g in a high-fat diet) counteracted lipid metabolism disturbances but had no significant effects on growth performance in rats [17].

### 3.2. Intestinal Morphology of LPS-Stimulated Mice

**Figure 3:**
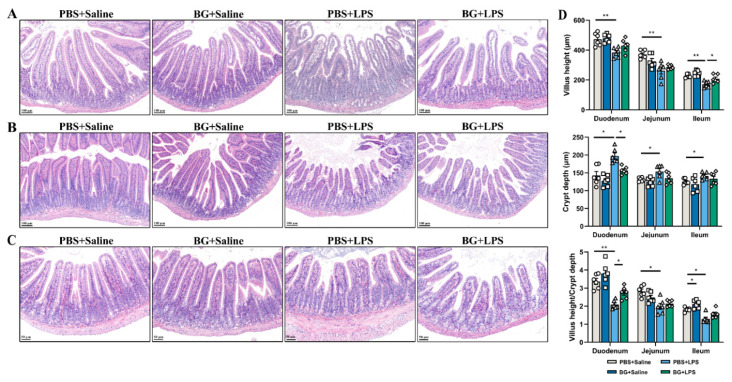
BG maintains intestinal morphology. H&E staining of sections of (**A**) duodenum (scale bar = 100 μm); (**B**) Jejunum (scale bar = 100 μm); (**C**) Ileum (scale bar = 50 μm); (**D**) Villus height, crypt depth, and the ratio of villus height and crypt depth of different sections of the small intestine. PBS+Saline, mice intragastric administered PBS and injection with saline; BG+Saline mice intragastric administered 1.5 g/kg BW BG and injection with saline; PBS+LPS, mice intragastrically administered PBS and injected with LPS; BG+LPS, mice intragastrically administered 1.5 g/kg BW BG and injected with LPS. Data are presented as mean ± SEM, *n* = 6. * and ** indicate significant difference (*p* < 0.05 and *p* < 0.01, respectively). An unpaired *t*-test was used for statistical analysis between two groups.

LPS injection significantly decreased the duodenum villus height and duodenum villus height to crypt depth ratio and increased the duodenum crypt depth (*p* < 0.05, Figure 3A,D). LPS stimulation significantly decreased the jejunum villus height and jejunum villus height to crypt depth in mice (*p* < 0.05, Figure 3B,D). BG administered at 1.5 g/kg BW attenuated the increase in duodenum crypt depth (*p* < 0.05, Figure 3A,D). BG administration (at 1.5 g/kg BW) did not help in the attenuation of the jejunum injuries (*p* > 0.05, Figure 3B,D). Under saline intraperitoneal injection, there were no significant differences in morphology or histological index of the duodenal structures between the PBS+Saline and BG+Saline groups (*p* > 0.05, Figure 3A,D). There were no significant differences in morphology or histological index of jejunal structures between the PBS+Saline and BG+Saline groups (*p* > 0.05, Figure 3B,D). Compared to the PBS+Saline group, LPS stimulation decreased the ileum villus height in PBS administered mice (*p* < 0.05, Figure 3C,D), however, when administered BG the ileum villus height decrease induced by LPS stimulation was attenuated (*p* < 0.05, Figure 3C,D). Under saline intraperitoneal injection, BG administration significantly increased the ileum villus height to crypt depth ratio in mice (*p* < 0.05). These results can be observed in Figure 3C.

### 3.3. Ultrastructure of Jejunal and Ileum Epithelial Cells in Mice Induced by LPS Challenging

**Figure 4:**
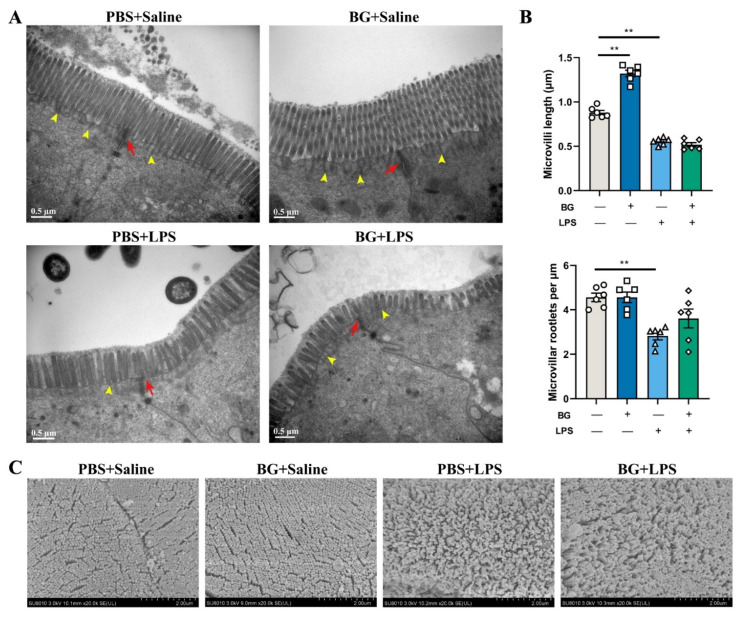
Attenuating effects of BG on the ultrastructure of jejunal epithelial cells in mice induced with LPS intraperitoneal injection. (**A**) Transmission electron microscope images of ileum epithelial cells (scale bar = 0.5 μm); (**B**) jejunum epithelial cells microvilli length and average microvilli rootlet densities. “+” and “−” mean with and without corresponding treatments, respectively. (**C**) Jejunum epithelial cells scanning electron microscope images (20,000×). Yellow arrowheads designate rootlet ends, and red arrows designate tight junction structures. PBS+Saline, mice administered PBS and injected with saline; BG+Saline mice administered 1.5 g/kg BW BG and injected with saline; PBS+LPS, mice administered PBS and injected with LPS; BG+LPS, mice administered 1.5 g/kg BW BG and injected with LPS. Data are presented as mean ± SEM, *n* = 6. ** indicate significant difference (*p* < 0.01). An unpaired *t*-test was used for statistical analysis between two groups.

**Figure 5:**
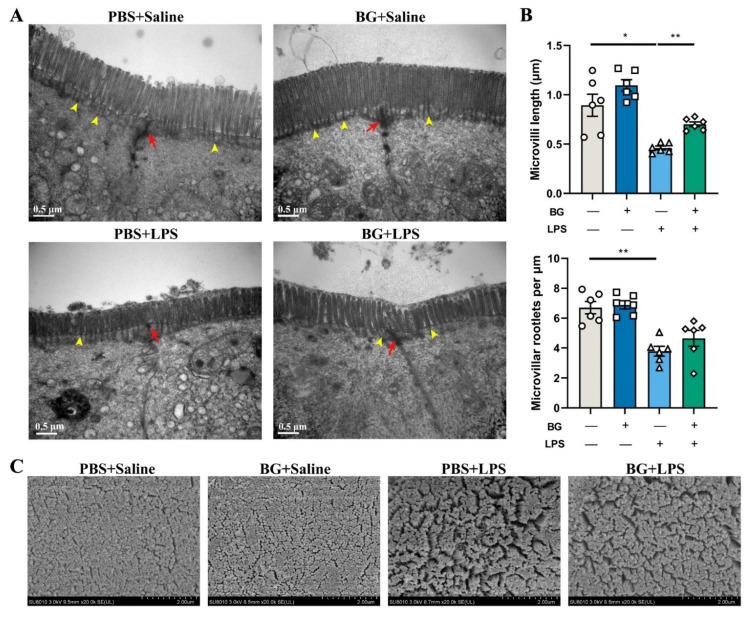
Attenuating effects of BG on ultrastructure of ileac epithelial cell in mice induced by LPS intraperitoneally injection. (**A**) transmission electron microscope images of ileum epithelial cells (scale bar = 0.5 μm); (**B**) ileum epithelial cells microvilli length and average microvilli rootlet densities; “+” and “−” mean with and without corresponding treatments, respectively; (**C**) ileum epithelial cells scanning electron microscope images (20,000×). Yellow arrowheads designate rootlet ends, and red arrows designate tight junction structures. PBS+Saline, mice administration with PBS, and injection with saline; BG+Saline mice administration with 1.5 g/kg BW BG and injection with saline; PBS+LPS, mice administration with PBS and injection with LPS; BG+LPS, mice administered 1.5 g/kg BW BG and injection with LPS. Data are presented as mean ± SEM, *n* = 6. * and ** indicate significant difference (*p* < 0.05 and *p* < 0.01, respectively). An unpaired *t*-test was used for statistical analysis between two groups.

The ultrastructure images of mice jejunal and ileal epithelial cells are shown in Figure 4 and Figure 5.

The observed results with TEM (Figure 4A and Figure 5A) showed that BG and PBS administration exhibited no harm to the jejunal and ileac epithelial cells. The cell gap and the tight junction structures were clear and complete. The microvilli of jejunal and ileac epithelial cells were well arranged. However, LPS stimulation, in the PBS-administered mice, induced the creation of cell gaps in the jejunum and ileal epithelium; the tight junction structures were broken and not clear. The microvilli became shorter and deficient. On the other hand, the BG gavage into the mice, attenuated the ileum brush border and tight junction structures damage caused by the LPS challenging. Nonetheless, there was no obvious relief effect on jejunum epithelial cells.

As shown in Figure 4B and Figure 5B, LPS stimulation decreased microvilli length and microvilli root density in the jejunum and ileum epithelial cells (*p* < 0.05). BG administration attenuated LPS-induced length shortening in the ileum epithelial cells (*p* < 0.05) but did not attenuate the reduction in microvilli root density in the jejunum and ileum (*p* > 0.05).

The observed results with SEM showed that the microvilli were intact, arranged and compact in the PBS and the BG groups injected with saline (Figure 4C and Figure 5C). LPS stimulation decreased the microvilli density of the jejunum and ileal epithelial cells in both PBS and BG in LPS stimulation. However, BG administration attenuated the microvilli loss.

### 3.4. BG Alleviates LPS-Induced Ileal Intestinal Barrier Function Injuries

**Figure 6:**
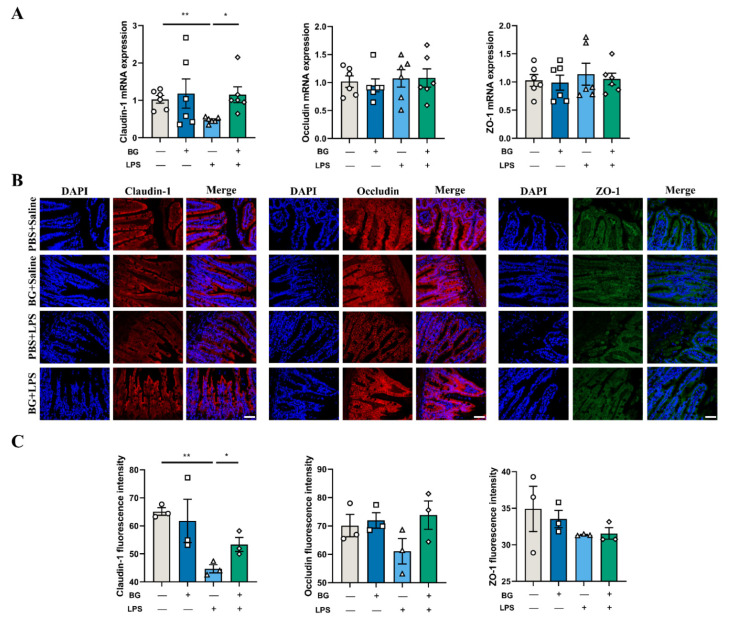
BG alleviated the reduction of tight junction protein expression in the ileum due to LPS induction. (**A**) The mRNA expression of tight junction proteins in the ileum. (**B**) The immunofluorescence images of claudin-1 (red), occludin (red), ZO-1 (green) and DAPI (blue) in the ileum. Scale bar: 50 μm. (**C**) The fluorescence intensity of claudin-1, occludin and ZO-1. PBS+Saline, mice administered PBS, and injected with saline; BG+saline mice administered 1.5 g/kg BW BG and injected with saline; PBS+LPS, mice administered PBS and injected with LPS; BG+LPS, mice administered 1.5 g/kg BW BG and injected with LPS. “+” and “−” mean with and without corresponding treatments, respectively. Data are presented as mean ± SEM. * and ** indicate significant difference (*p* < 0.05 and *p* < 0.01, respectively). An unpaired *t*-test was used for statistical analysis between two groups.

As shown in Figure 6A, BG administration attenuated the ileum mRNA expression of TJ protein claudin-1 reduction by LPS injection (*p* < 0.05). No significant differences were observed in the mRNA expression of tight junction protein ZO-1 and occludin (*p* > 0.05). Immunofluorescence was used to further study the effects of BG on TJ protein location and expression after LPS injection (shown in Figure 6B). LPS challenging reduced the expression of claudin-1 in the ileum, while the expression of claudin-1 was improved by BG administration (*p* < 0.05). Collectively, these results indicate that BG administration could significantly improve the expression of TJ proteins in the ileum.

### 3.5. BG Reduce Ileal Inflammatory Response

**Figure 7:**
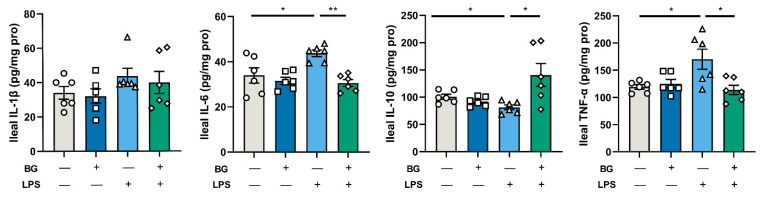
BG alleviated LPS-induced ileal intestinal inflammation response. Data are presented as mean ± SEM, *n* = 6. “+” and “−” mean with and without corresponding treatments, respectively. * and ** indicate significant difference (*p* < 0.05 and *p* < 0.01, respectively). An unpaired *t*-test was used for statistical analysis between two groups.

As shown in Figure 7, LPS injection decreased the ileal IL-10 level and increased the IL-6 and TNF-α levels in mice intragastrically administered PBS, however, this was remitted in the BG intragastric administered mice (*p* < 0.05). Moreover, there were no significant differences in the level of IL-1β between the groups (*p* > 0.05).

### 3.6. Inflammatory Responses of LPS-Stimulated Mice

**Figure 8:**
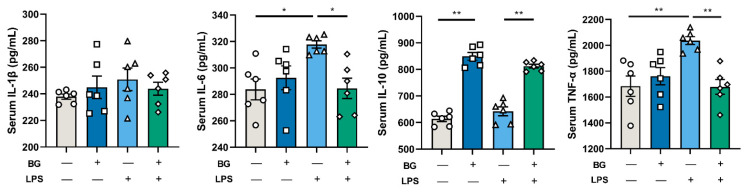
BG administration regulated the inflammatory responses of LPS-stimulated mice. Data are presented as mean ± SEM, *n* = 6. “+” and “−” mean with and without corresponding treatments, respectively. * and ** indicate significant difference (*p* < 0.05 and *p* < 0.01, respectively). An unpaired *t*-test was used for statistical analysis between two groups.

As shown in Figure 8, no significant differences were observed in the levels of IL-1β and IL-10 in serum between mice injected with LPS or saline. However, LPS injection significantly increased TNF-α and IL-6 in the serum of mice intragastrically administered with PBS (*p* < 0.05). Nonetheless, BG alleviated their increase (*p* < 0.05, *p* < 0.01) and increased the levels of serum IL-10 (*p* < 0.01). Mice from the BG intragastric administration group, compared with the mice with intragastric administration of PBS, showed significantly increased levels of serum IL-10 (*p* < 0.01), while the levels of IL-1β exhibited no significant differences (*p* > 0.05).

### 3.7. BG Alleviated Intestinal Inflammatory Response Induced by LPS-Stimulated via JNK Signaling Pathways

**Figure 9:**
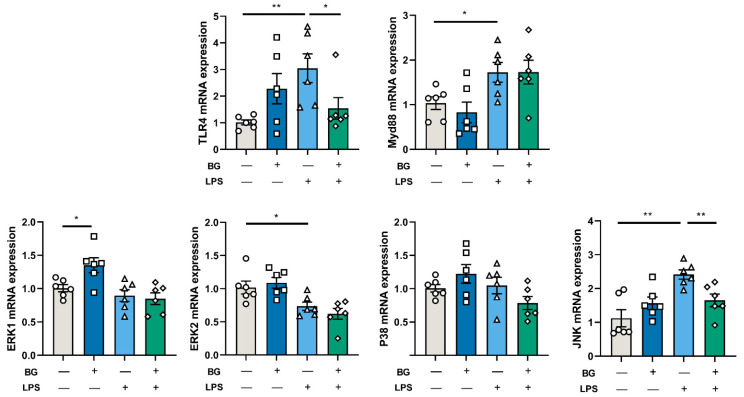
BG alleviated the mRNA expression increasing of TLR4/Myd88/JNK signal pathway in the ileum induced by LPS stimulation. Data are presented as mean ± SEM, *n* = 6. “+” and “−” mean with and without corresponding treatments, respectively. * and ** indicate significant difference (*p* < 0.05 and *p* < 0.01, respectively). An unpaired *t*-test was used for statistical analysis between two groups.

To investigate the potential signaling pathway involved in releasing intestinal inflammatory cytokines, mRNA expression of the mitogen-activated protein kinases (MAPKs) and pattern recognition receptors (PRRs)were determined. As shown in Figure 9, BG administration attenuated the ileum mRNA expression of TLR4 and JNK increasing induced by LPS injection (*p* < 0.05). No significant differences were observed in the MAPKs signal path P38 (*p* > 0.05). LPS stimulation increased Myd88 and decreased the mRNA expression of ERK2 (*p* < 0.05). Moreover, in the BG+Saline group, BG administration increased the mRNA expression of ERK1 (*p* < 0.05).

## 4. Discussion

Intestinal barrier function plays a critical role in animal health [22]. When the intestinal barrier is injured, the chances of harmful substances and pathogenic microorganisms trespassing the intestinal tissue into the bloodstream are increased, leading to a series of disorders [23]. Butyrate has many biological functions and among them, the ability to inhibit an intestinal inflammation response [24]. However, free butyric acid has an unpleasant smell and a fast absorption in the foregut, which limits its use. As a derivative of butyric acid, BG overcomes these shortcomings. This paper has presented two different studies that investigated whether BG has a potential protective effect on intestinal inflammation and barrier dysfunction in LPS-stimulated mice. In the first study, we demonstrated that 1.5 g/kg BW BG could alleviate ileal inflammation and maintain duodenum and ileum morphology and structure (shown in Figures S2 and S3). In the second study, we explored the underlying mechanisms of BG in alleviating intestinal inflammation using the identified BG optimal dose for the first study.

The small intestinal mucosa is the primary part where digestion and absorption of nutrients take place, it also prevents pathogens and toxic substances that are present in the intestinal lumen from attacking the intestinal tissue and translocating into the host system. The intestinal villus height, crypt depth and the ratio of villus height/crypt depth are critical indicators that reflect the structural integrity of the intestinal tract [25]. The results showed that LPS stimulation induced a reduction in villus height, an increase in crypt depth and an overall reduction in the ratio of villus height/crypt depth (shown in Figure S2), which indicates that LPS stimulation disrupted the intestinal morphology and structure. These results are consistent with previous studies [26]. Intestinal morphology analysis showed that 0.5–2.5 g/kg BW BG administration alleviated duodenum morphology injury. Among them, BG dosage at 1.5 g/kg BW could alleviate duodenum and ileum morphology injury (shown in Figure S2). Hou et al. showed that butyrin glycerides alleviated the ratio of villus height/crypt depth decreasing in the ileum of piglets caused by acetic acid stimulation, which was similar to our results [27]. Data of pro- and anti-inflammatory cytokine mRNA expression showed that BG attenuated the increase in mRNA expression of TNF-α in the ileum. It was reported that an increase in IL-10 may inhibit the secretion of pro-inflammatory factors [28]. In our study, the mice gavaged with BG (1.5 g/kg BW BG) and injected with LPS, showed increased mRNA expression of IL-10 in the jejunum and ileum (shown in Figure S3). These results imply that 1.5 g/kg BW BG administration can promote the secretion of immunomodulatory cytokines in the jejunum and ileum, which improve the intestinal immune response against intestinal challenges such as LPS, reducing the inflammatory response. Previous findings reported by Fu et al. and Chen et al. suggested that butyrate could attenuate the inflammatory response in the ileum and colon, which is consistent with our experimental results [29,30]. Thus, 1.5 g/kg BW BG was selected as the optimal dose for the second experiment to explore the underlying mechanism of BG in alleviating intestinal inflammation and barrier dysfunction.

LPS is a component of the outer membrane of Gram-negative bacteria [31]. After entering the blood circulation, LPS activates the immune response of the body, resulting in the release of a large number of inflammatory cytokines [32]. IL-1β is a pro-inflammatory cytokine that can activate macrophages and epithelial cells and respond to infection and injury [33]. IL-6 is a pleiotropic cytokine that plays a crucial part in the acute phase response and affects intestinal permeability by regulating tight junction structures [34]. IL-10 is the primary immunomodulatory cytokine in the intestine and can attenuate the production of inflammatory cytokines. TNF-α has the central role of initiating the inflammatory responses [33,35]. Administering 1.5 g/kg BW BG attenuated intestinal and body inflammatory responses by LPS stimulation in mice (shown in Figure 7 and Figure 8).

Most nutrient digestion and absorption occur in the jejunum and ileum [36]. The apex of intestinal epithelial absorptive cells contain thousands of microvilli, which form the brush border and increase the available surface area for absorption of nutrients [37]. Microvilli length is generally 1–2 μm, depending on intestinal epithelial cell differentiation status and region [38]. Healthy and fully differentiated intestinal epithelial absorptive cells have a distinct brush border that are highly ordered and uniform [39]. The brush border is the physical electrostatic barrier that reduces the contact between pathogens and the intestinal epithelium [40]. The pro-inflammatory cytokines disrupt the structural integrity of the brush border. Decreased length and rootlet density of ileum microvilli have been observed in patients with Crohn’s disease (CD), a form of inflammatory bowel disease [41]. If there is damage to the brush border, the damage to the brush border increases the possibility that harmful bacteria are internalized in the gut. The invasion of pathogens further damages the brush border resulting in the inflammatory response of the body and leading to secondary damage of TJ structures [4,42]. With TEM observation on the jejunum and ileum epithelial cells, we found that LPS stimulation caused damage to brush border and TJ structures. BG administration alleviated LPS-induced microvilli height reduction and protected microvilli arrangement and TJ structures (shown in Figure 4 and Figure 5). Furthermore, BG increased ileum villus height in mice under normal conditions, which seems to indicate that BG has better effects on the ileum (shown in Figure 5). Thus, we selected the ileum as the primary object for following research to find how BG affect intestinal barrier function and regulate intestinal inflammatory responses.

TJ are one of the intestinal epithelial cell connections that provide physical barrier limits to intestinal contents crossing into deeper tissue [43]. The main TJ composition of the intestinal epithelium are claudins, occludin and ZO. Occludin and claudins are two tetraspanin membrane proteins that regulate intestinal permeability and establish the intestinal barrier. Furthermore, the function of ZO proteins is to bind the transmembrane proteins to the actin cytoskeleton [42]. Intestinal TJ disruption causes the penetration of harmful molecules within the lumen into the tissue, and therefore disturbs the intestinal immune system [44]. LPS stimulation resulted in claudin-1 mRNA expression decreasing in the ileum of mice, which indicated that LPS stimulation caused certain injury to the TJ structures. Immunofluorescence results showed that BG administration attenuated the decrease in the expression of claudin-1 (shown in Figure 6B). Those results are consistent with Nguyen et al. and Lee et al., who reported that monobutyrin improves intestinal barrier function in high-fat diet fed animals [17,19]. Those results suggest that BG has a protective effect on LPS-induced TJ impairment under pathological conditions.

In summary, LPS stimulation leads to a dramatic inflammatory response and production of inflammatory cytokines in the intestine. Inflammatory cytokines damage the normal physiological morphology, ultrastructure and TJ structures of the intestine. In the LPS-stimulated pathological state, BG protect the intestine from damage and alleviate the inflammatory response. To further explore the underlying mechanisms of BG in protecting intestinal barrier function, we analyzed the mRNA expression of MAPKs signaling pathways.

LPS was able to activate PRRs, especially for TLR4. TLR4 activation turns on a series of downstream signaling pathways such as NF-κB and MAPKs which result in the release of inflammatory factors and promotion of an intestinal inflammatory response in the intestine [45]. PRRs widely exist in intestinal epithelial cells (IECs). IECs recognize different microorganisms and pathogens via PRRs to trigger corresponding immune responses. The myeloid differentiation primary response gene88 (Myd88) is a universal adaptor protein that plays a vital role in the TLR4-mediated immune response, including Myd88-dependent (which results in the activation of MAPKs) and Myd88-independent pathways [45]. In the MAPKs signal pathway, ERK1 and ERK2 are preferentially activated by stimulating growth and proliferation factor, while the activation of P38 and JNK signal pathways are related to many factors, especially stress and toxins [46,47]. In this study, LPS stimulation increased the mRNA expression of TLR4, Myd88 and JNK in the ileum of mice. These together lead to an increase in the pro-inflammatory cytokines IL-6 and TNF-α and a decrease in the immunomodulatory cytokine IL-10 in the ileum (shown in Figure 7). Supplementation of BG under LPS injection conditions did not alleviate the increased mRNA expression of Myd88, but it relieved the increase of JNK mRNA expression (shown in Figure 9). It is possible that BG administration inhibited the activation of JNK, thereby alleviating the expression of ileum inflammatory cytokines (IL-6 and TNF-α). In addition, LPS stimulation decreased the mRNA expression of ERK2 mRNA in the ileum. Under normal conditions, BG administration increased the mRNA expression of ERK1. However, whether BG is associated with the regulation of the cell cycle and proliferation requires further investigation.

In the present study, BG administration reduced the production of intestinal inflammatory cytokines via inhibition of JNK expression. As a consequence, BG declined the inflammatory cytokines damaging effects on the physiology, morphology and structure of the TJs. BG protected the microvilli arrangement and length of the brush border, thereby promoting the physical electrostatic barrier of the intestinal epithelium. 

## 5. Conclusions

BG administration alleviated intestinal morphological and ultrastructure injury and protected the expression of TJ proteins. Moreover, BG attenuated the body and intestinal inflammatory responses induced by LPS. BG alleviation of ileum intestinal inflammation might be associated with the MAPKs signaling pathway. This needs to be verified in further experiments. Finally, BG could maintain intestinal health and is a promising prospect for future applications. 

## Figures and Tables

**Table 1:** Target Gene primer sequences designed for RT-qPCR.

Gene	Sequences	Gen Bank Accession Number of mRNAs
IL-1β	F: TCGCAGCAGCACATCAACAGAG R: AGGTCCACGGGAAAGCACAGG	NM_008361
IL-6	F: CTGCAAGAGACTTCCATCCAG R: AGTGGTATAGCAGGTCTGTTGG	NM_031168
IL-10	F: TTCTTTCAAACAAAGGACCAGC R: GCAACCCAAGTAACCCTTAAAG	NM_010548
TNF-α	F: ATGTCTCAGCCTCTTCTCATTC R: GCTTGTCACTCGAATTTTGAGA	NM_001278601
TLR4	F: GCCTTTCAGGGAATTAAGCTCC R: GATCAACCGATGGACGTGTAAA	NM_021297
Myd88	F: ATCGCTGTTCTTGAACCCTCG R: CTCACGGTCTAACAAGGCCAG	NM_010851
ERK1	F: ACCACATTCTAGGTATCTTGGGT R: AGTTTCGGGCCTTCATGTTAAT	NM_011952
ERK2	F: TTGCTTTCTCTCCCGCACAAA R: AGAGCCTGTTCAACTTCAATCC	NM_001038663
P38	F: GAGAAGATGCTCGTTTTGGACT R: GGACTGGTCATAAGGGTCAGC	NM_011951
JNK	F: AGCAGAAGCAAACGTGACAAC R: GCTGCACACACTATTCCTTGAG	NM_016700
Claudin-1	F: GGGGACAACATCGTGACCG R: AGGAGTCGAAGACTTTGCACT	NM_016674
Occludin	F: TTGAAAGTCCACCTCCTTACAGA R: CCGGATAAAAAGAGTACGCTGG	NM_008756
ZO-1	F: GCCGCTAAGAGCACAGCAA R: TCCCCACTCTGAAAATGAGGA	NM_009386
β-actin	F: TATGCTCTCCCTCACGCCATCC R: GTCACGCACGATTTCCCTCTCAG	NM_007393

## Data Availability

The data used to support the findings of this study are available from the corresponding author upon request.

## Supplementary Materials

The following supporting information can be downloaded at: https://www.mdpi.com/article/10.3390/nu14193991/s1, Figure S1: Changes of body weight during the optimal dose of gavage administration experiment; Figure S2: Effects of BG administered at different doses on intestinal morphology of LPS-stimulated mice; Figure S3: Effects of BG on inflammatory and anti-inflammatory cytokines mRNA expression of jejunum and ileum in LPS-stimulated mice.
