# Supplementary material for: Butyrate Glycerides Protect against Intestinal Inflammation and Barrier Dysfunction in Mice
Source: Nutrients. 2022 Sep 26;14(19):3991. doi: 10.3390/nu14193991 (PMC9570839; doi:10.3390/nu14193991)
Supplement: Supplementary file 1 [file nutrients-14-03991-s001.zip › nutrients-1911320-supplementary.pdf]

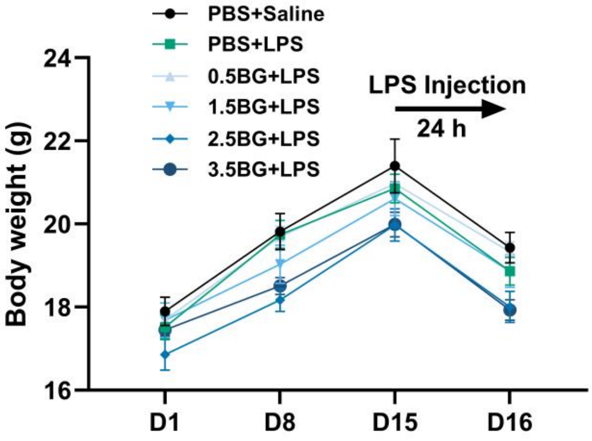

Figure S1. Changes of body weight during the optimal dose of gavage administration experiment.

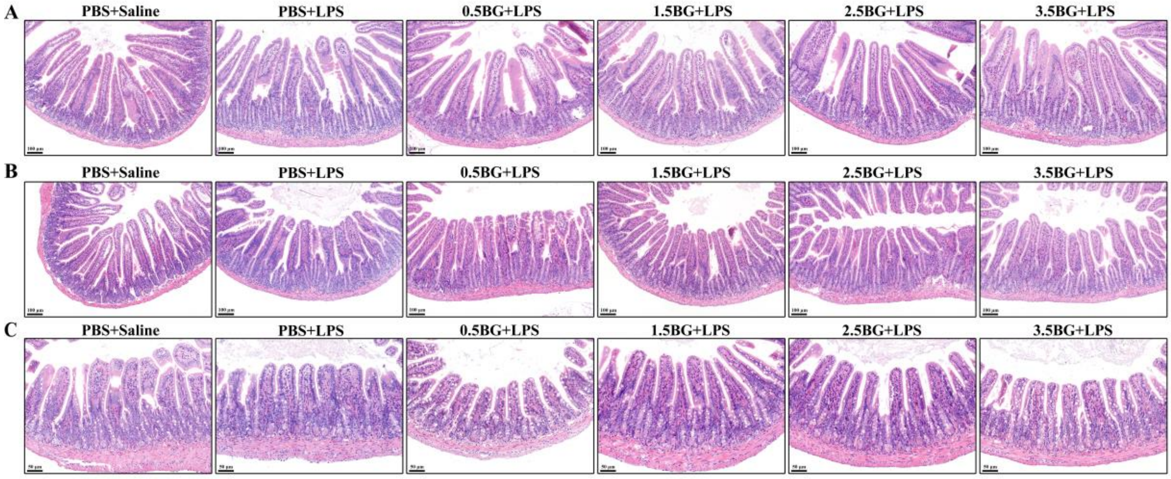

D

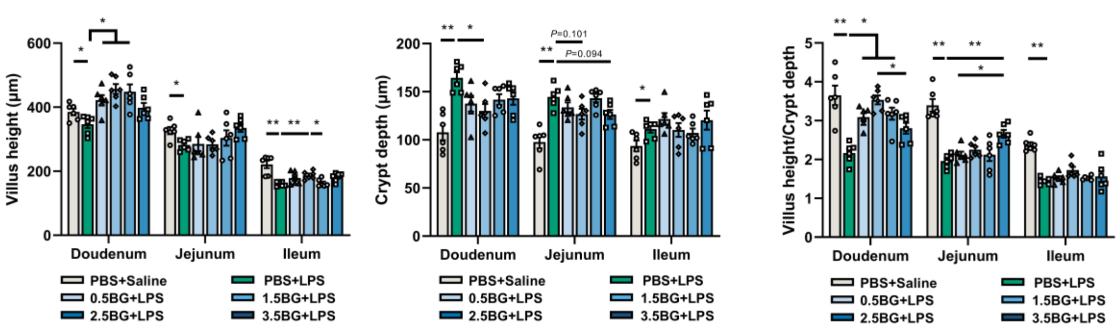

Figure S2. Effects of BG administered at different doses on intestinal morphology of LPS-stimulated mice. (A) duodenum (Scale bar = 100 μm); (B) Jejunum (Scale bar = 100 μm); (C) Ileum (Scale bar = 50 μm); (D) Villus height, crypt depth, and the ratio of villus height and crypt depth of different sections

of the small intestine. Data were represented as mean ± SEM, *n*=6. * and ** indicate significant difference (p<0.05 and p<0.01).

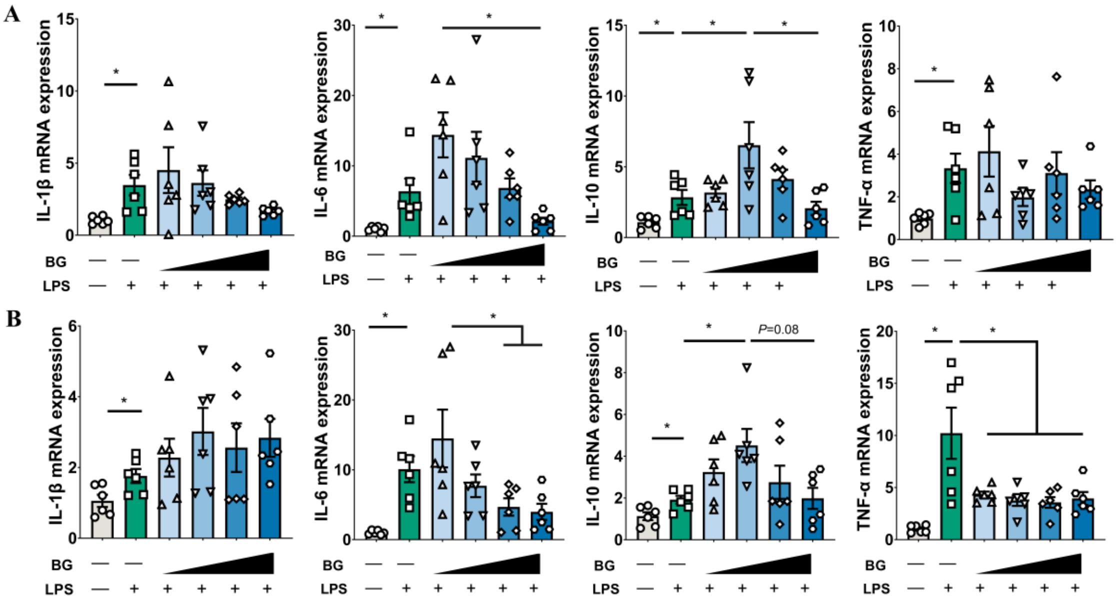

Figure S3. Effects of BG on inflammatory and anti-inflammatory cytokines mRNA expression of jejunum and ileum in LPS-stimulated mice. (A) The inflammatory and anti-inflammatory cytokines mRNA expression of jejunum (B) The inflammatory and anti-inflammatory cytokines mRNA expression of ileum. Data were represented as mean ± SEM, *n*=6. * indicate significant difference (p<0.05).
